# Menopausal hormone therapy and primary liver cancer: long-term follow-up of the women’s health initiative randomized trials

**DOI:** 10.1038/s41416-026-03621-9

**Published:** 2026-09-16

**Authors:** Margaret S. Pichardo, Aaron K. Aragaki, JoAnn E. Manson, Kathy Pan, Su Yon Jung, Thomas E. Rohan, Reed Mszar, Rowan T. Chlebowski

**Affiliations:** 1 Department of Surgery, University of Pennsylvania, Philadelphia, PA, USA.; 2 Division of Public Health Sciences, Fred Hutchinson Cancer Center, Seattle, WA, USA.; 3 Department of Medicine, Brigham and Women’s Hospital, Harvard Medical School, Boston, MA, USA.; 4 Kaiser Permanente Southern California, Downey, CA, USA.; 5 Translational Sciences Section, School of Nursing, Department of Epidemiology, Fielding School of Public Health, Jonsson Comprehensive Cancer Center, University of California, Los Angeles, CA, USA.; 6 Department of Epidemiology and Population Health, Albert Einstein College of Medicine, Bronx, NY, USA.; 7 Department of Chronic Disease Epidemiology, Yale School of Public Health, New Haven, CT, USA.; 8 The Lundquist Institute, Torrance, CA, USA.

## Abstract

**BACKGROUND::**

Long-term results on liver cancer incidence and mortality are reported from two Women’s Health Initiative randomized trials evaluating menopausal hormone therapy.

**METHODS::**

16,608 postmenopausal women with a uterus were randomized to conjugated equine estrogen (CEE) 0.625 mg/d plus medroxyprogesterone acetate (MPA) 2.5 mg/d or placebo and 10,739 women with hysterectomy were randomized to 0.625 mg/d of CEE-alone or placebo.

**RESULTS::**

After nearly 24 years follow-up and 74 incident liver cancers, neither CEE-alone nor CEE plus MPA influenced liver cancer development (CEE-alone vs placebo (11 vs 15 cancers; hazard ratio [HR] 0.75; 95% CI, 0.34–1.63; CEE plus MPA vs placebo (30 vs 18 cancers; HR 1.63; 95% CI, 0.91–2.92). CEE plus MPA did not significantly influence liver cancer mortality: 27 vs 22 deaths (HR 1.18; 95% CI, 0.67–2.07). In subgroup analyses, CEE plus MPA vs placebo was associated with more liver cancers among women aged 50–59 years (10 vs 0 cancers (P-trend 0.01) and prior oral contraceptive users (HR 4.30, 95% CI, 1.46–12.72; P-interaction 0.01).

**CONCLUSIONS::**

These findings indicate no overall influence of menopausal hormone therapy on liver cancer incidence or mortality although a possible increased risk with CEE plus MPA in select subgroups warrants further investigation.

**TRIAL REGISTRATION:**

clinicaltrials.gov Identifier: NCT00000611

## BACKGROUND

There are significant gender differences in the incidence and outcome of liver cancer [1]. Compared with men, the incidence and mortality of liver cancer in women are substantially lower with 14,020 new cases in women versus 28,220 in men in 2025 [2]. Notably, the gap narrows after menopause, suggesting a role for hormonal or reproductive factors [3]. For over two decades, alterations in estrogen signaling pathways have been postulated to influence the occurrence and development of hepatocellular carcinoma [3–5]. However, observational study evidence on estrogen exposure and hepatocellular carcinoma risk is inconsistent. In the US Liver Cancer Pooling Project among 799,500 women with 203 hepatocellular carcinomas, bilateral oophorectomy and menopausal hormone therapy were associated with higher liver cancer risk (hazard ratio [HR], 1.35; 95% CI, 1.01–1.81), though the latter attenuated after adjusting for oophorectomy [6]. A meta-analysis of 1795 cases reported higher risk with oophorectomy, but lower risk with menopausal hormone therapy (RR, 0.60; 95% CI, 0.37–0.96) [5]. Thus, the influence of menopausal hormone therapy on liver cancer is uncertain. To our knowledge, no randomized trials have reported on this question, a gap which is addressed here.

## METHODS

The Women’s Health Initiative (WHI, Clinical Trial# NCT00000611) included two randomized, double-blind, placebo-controlled hormone therapy trials conducted in 40 U.S. clinical centers from 1993 to 1998 to evaluate menopausal hormone therapy. The design and conduct of the trials have been described previously [7–9]. Details of the public’s involvement in the design and conduct of the WHI trial can be found here [10]. For both trials, the primary protocol-defined monitoring outcome for benefit with coronary heart disease and the primary monitoring outcome for harm was breast cancer. Thus, breast cancer incidence was a primary study outcome [11]. Secondary outcomes included hip fractures, stroke, venous thromboembolism, and mortality across both trials. The CEE plus MPA trial additionally included colorectal and endometrial cancers as secondary outcomes. Trial eligibility included postmenopausal women aged 50–79 years who provided written informed consent. Major exclusions included prior breast cancer, anticipated survival < 3 years, other invasive cancer within 10 years. The sample sizes were chosen to provide approximately 80% power to detect effects on the primary outcomes; the present secondary analysis used all randomized participants [12].

Postmenopausal women with a uterus (n = 16,608) were randomized to conjugated equine estrogen (CEE) 0.625 mg/d plus medroxyprogesterone acetate (MPA) 2.5 mg/d or placebo; women with hysterectomy (n = 10,739) were randomized to CEE-alone or placebo. Treatment assignment (1:1 allocation) was performed through a centralized database function that verified eligibility and assigned participants using a randomized permuted-block algorithm stratified by clinical center, age group, and hysterectomy status, with randomly varying block sizes. Before randomization, hormone therapy participants completed a placebo run-in period; adherence of less than 80% was considered inadequate. The intervention was self-administered as once daily oral tablets, hormone therapy or matching placebo, with temporal holds for safety if mammography screening was overdue. After randomization, adherence was assessed by pill counts at follow-up visits. Blinding was maintained using matching active and placebo tablets, with all participants taking 1 tablet daily; unblinding was permitted only when necessary for safety. Participants continued usual care with their own physicians augmented with protocolized specified annual mammography and clinical exams applied uniformly across arm [7, 8].

Adverse events were prospectively monitored throughout follow-up, including vaginal bleeding, endometrial abnormalities, venous thromboembolism, breast cancer, and other serious adverse experiences, with regular review by the Data and Safety Monitoring Board. The CEE plus MPA trial was stopped early in 2002 after 5.6 years because overall risks were judged to outweigh benefits. The CEE-alone trial was stopped early in 2004 after 7.2 years because of increased stroke risk and no clear coronary heart disease benefit.

Clinical outcome ascertainment was monthly through 2005 with subsequent updates annually. Follow-up beyond the protocol-specified end date for incidence required written re-consent. All incident cancers, including liver cancers, were confirmed by central medical record review by reviewers blinded to randomization assignment. Participants’ own physicians directed liver cancer therapy. Deaths and cause of death were verified by central medical record or death certificate review while serial National Death Index queries through 2023 provide near complete (98%) information on mortality and cause of death independent of re-consent status [13].

The current liver cancer analyses were not protocol-specified. Outcomes included incidence of and mortality from primary liver cancer, analyzed separately by trial group. Analyses followed the intention-to-treat principle. Follow-up time continued until December 31, 2023, first cancer, death, or loss to follow-up. Cox proportional hazards models estimated hazard ratios (HRs) and 95% confidence intervals (CIs), with baseline hazard functions stratified by age group, dietary modification trial randomization, and study period. Two-sided stratified log-rank tests were used to test for differences between the intervention and control groups, with P ≤ 0.05 considered statistically significant. Temporal patterns were assessed using Kaplan-Meier curves with cumulative HRs (95% CIs) under proportional hazards assumptions [14]. Given the limited number of events, subgroup analyses were restricted to the CEE plus MPA trial and, where appropriate, supplemented with sensitivity analyses to aid interpretation [15]. For interpretative purposes, an overall HR for liver cancer was computed across the two HT trials using an inverse-variance weighted average of the trial-specific log-HRs, analogous to a fixed-effect meta-analysis.

Analyses were conducted using SAS version 9.4 and R version 4.4. Statistical code used to estimate the primary results is available from the corresponding author upon reasonable request.

## RESULTS

For each trial, randomization group characteristics were generally well balanced at baseline (Table 1), although bilateral oophorectomy was less common in CEE-alone vs placebo group women (39.5% vs. 42%, P = 0.01). Trial cohorts differed, with the CEE-alone trial including women with higher prevalence of obesity, diabetes or hyperglycemia, and younger age at menopause, while the CEE plus MPA trial had higher alcohol and prior oral contraceptive use. Self-reported liver disease was rare (~ 2.2–2.5%). A total of 74 incident liver cancers were identified through December 31, 2023: 26 in the CEE-alone trial and 48 in the CEE plus MPA trial.

**Fig. 1:**
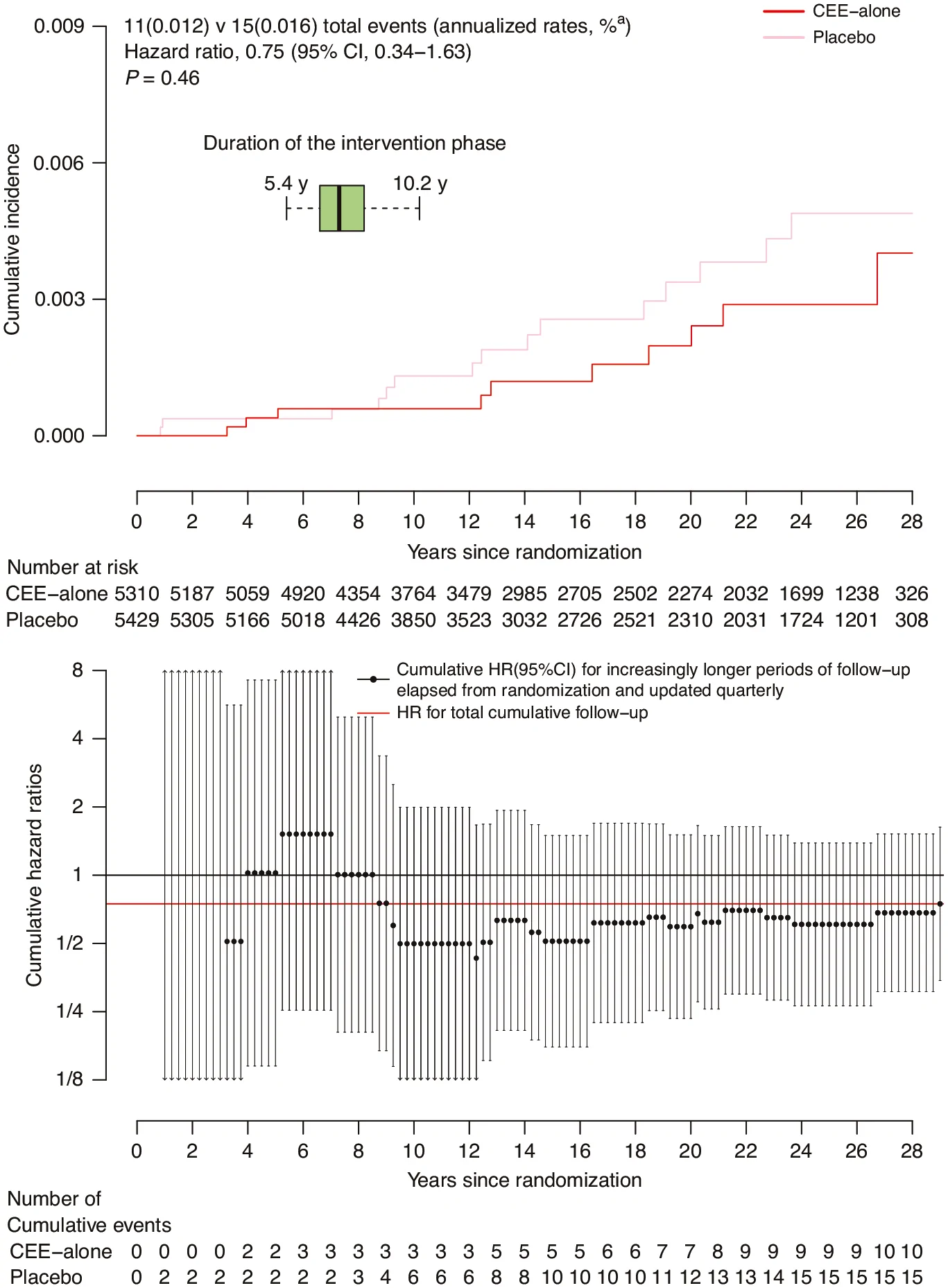
Kaplan–Meier estimates, complemented by cumulative hazard ratios, for liver cancer incidence in the CEE-alone trial. Cumulative HRs (95% CI) were computed under the proportional hazards assumption, using increasingly longer cumulative follow-up elapsed from random assignment; Cox regression model was stratified by age group, randomization status in the dietary trial, and study phase (time-dependent). Red line indicates the estimated HR = 0.75 for total cumulative follow-up, essentially a weighted average of period-specific HRs: HR = 1.52 (95% CI: 0.25–9.09) and HR = 0.63 (95% CI: 0.26–1.52) for the intervention and postintervention periods, respectively. There was no evidence of nonproportionality (P = 0.40). ^a^ Annualized rates were calculated by dividing the total number of events by total follow-up time in years and expressed as a percentage. CEE conjugated equine estrogens, HR hazard ratio.

In the CEE-alone trial, median cumulative follow-up for liver cancer incidence was 16.3 years. During the intervention phase, only five liver cancers occurred (HR, 1.52; 95% CI, 0.25–9.09). Through cumulative follow-up, CEE-alone was not associated with liver cancer incidence (11 cancers [0.012% per year] vs 15 cases [0.016% per year]); HR, 0.75; 95% CI, 0.34–1 .63). Results were unchanged after adjustment for bilateral oophorectomy status. No evidence of violation of the proportional hazard assumption was observed (P = 0.40; Fig. 1).

**Fig. 2:**
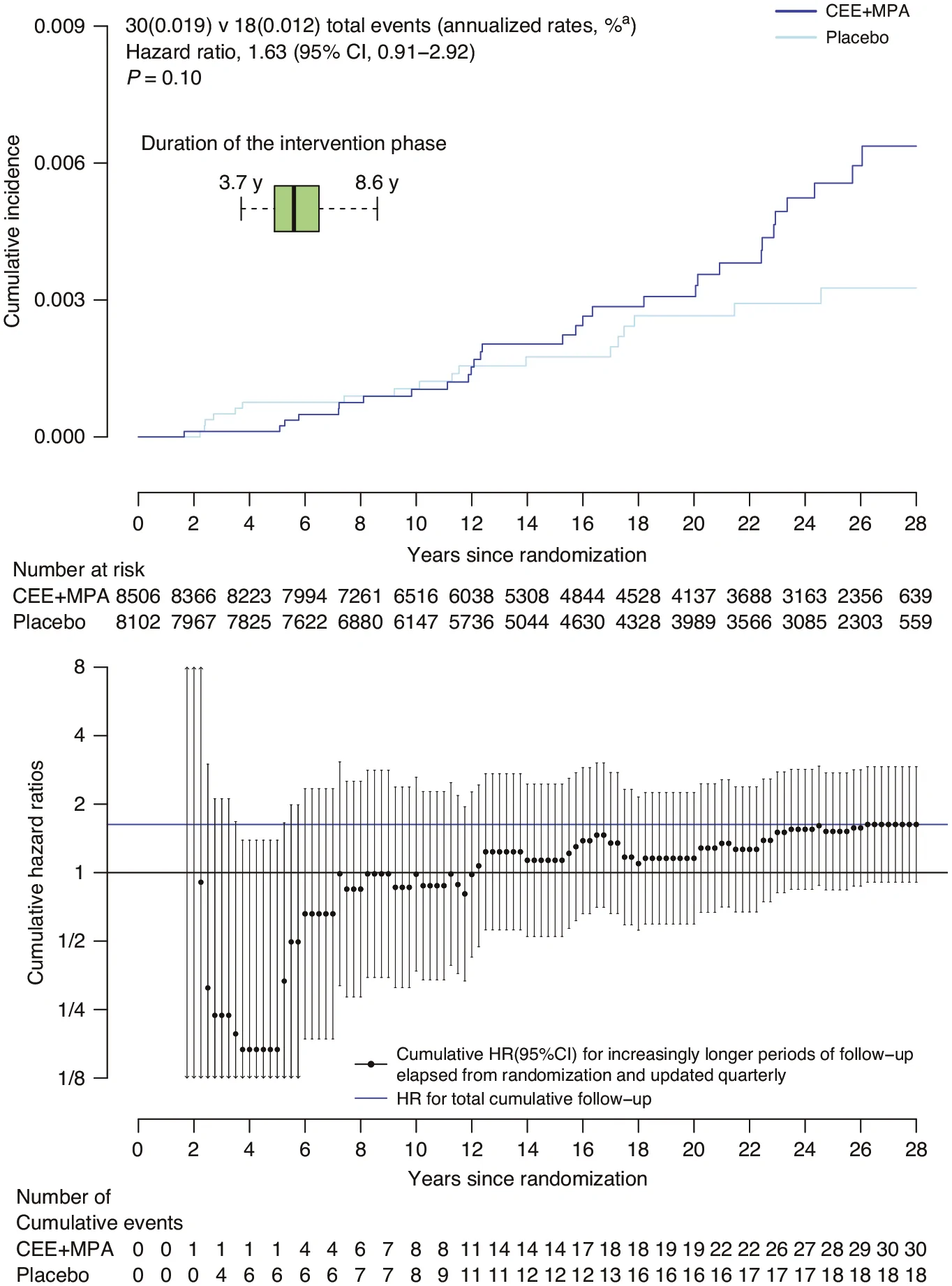
Kaplan–Meier estimates, complemented by cumulative hazard ratios, for liver cancer incidence in the CEE + MPA trial. Blue line indicates the estimated HR = 1.63 for total cumulative follow-up, essentially a weighted average of period-specific HRs: HR = 0.66 (95% CI: 0.19–2.34) and HR = 2.11 (95% CI: 1.06–4.18) for the intervention and postintervention periods, respectively. There was significant evidence of non-proportionality (P = 0.049). See Fig. 1 legend for additional details. ^a^ Annualized rates were calculated by dividing the total number of events by total follow-up time in years and expressed as a percentage. CEE conjugated equine estrogens, HR hazard ratio, MPA medroxyprogesterone acetate.

In the CEE + MPA trial, cumulative follow-up for liver cancer incidence was 19.5 years. During the intervention phase, 10 liver cancers occurred. Over cumulative follow-up, liver cancer incidence did not differ significantly between the CEE plus MPA and placebo group (30 cancer [0.019% per year vs 18 cancers [0.012% per year]; HR 1.63; 95% CI, 0.91–2.92; Fig. 2). Evidence of non-proportional hazards was observed (P = 0.049), with HRs for CEE plus MPA vs placebo elevated in the post-intervention phase (HR, 2.11; 95% CI, 1.06–4.18).

After a median cumulative follow-up for liver cancer mortality of 23.7 years in the CEE-alone trial and 24.7 years in the CEE plus MPA trial, no significant effects on liver cancer specific mortality were observed. For CEE-alone, there were 14 vs 12 deaths (HR, 1.18; 95% CI, 0.55–2.55), while for CEE plus MPA, there were 27 vs 22 deaths (HR, 1.18; 95% CI, 0.67–2.07).

In stratified analyses of the CEE plus MPA trial (Supplementary Fig. 1), higher risk was observed among prior oral contraceptive users (HR, 4.30; 95% CI, 1.46–12.72, P-interaction=0.01). Excess cases were also observed among women aged 50–59 years (10 cases in the intervention group vs 0 in placebo, P-trend=0.01), and essentially unchanged after additional adjustment for oral contraceptive use, with excess cases observed among both younger women who reported prior oral contraceptive use (6 vs 0) and those that did not (4 vs 0). No consistent effect modification was seen by smoking, body mass index, or prior menopausal hormone therapy. The association among current drinkers was somewhat stronger (HR, 2.02; 95% CI, 0.98–4.15), although the interaction test was not statistically significant (P-interaction = 0.50). Exploratory analyses further subdivided current drinkers into those reporting <1 drink/week (7 vs 5 cases; HR, 1.30; 95% CI, 0.41–4.09) and ≥1 drink/week (16 vs 6 cases; HR, 2.70; 95% CI, 1.06–6.92), but the corresponding test for interaction remained non-significant (P-interaction = 0.50). There were too few cases in the CEE- alone trial to warrant subgroup analyses. Averaging the HT vs placebo comparisons across the two trials, the overall HR for liver cancer incidence (HR, 1.23; 95% CI, 0.77–1.96) and overall HR for liver cancer mortality (HR, 1.18; 95% CI, 0.75, 1.86) were not significant. These estimates can be interpreted as applying to a WHI-like postmenopausal population, with approximately 60% having an intact uterus and 40% having had a hysterectomy.

Tumor characteristics were not completely available, particularly for cases ascertained exclusively from death reports. Characteristics were available for 17 of 26 liver cancers in the CEE-alone trial and 32 of 48 in the CEE + MPA trial. Among cases with available data, hepatocellular carcinoma was the most common histologic type. Given these small numbers and incomplete ascertainment, histology-specific comparisons were descriptive and did not support formal inference (Supplementary Table 1).

## DISCUSSION

In this long-term follow-up of the WHI hormone therapy trials, neither CEE-alone nor CEE plus MPA significantly influenced liver cancer incidence or liver cancer specific mortality over 25 years.

However, in the CEE + MPA trial, evidence of non-proportional hazards was observed, with liver cancer incidence higher in the postintervention period. In subgroup analyses, CEE plus MPA vs placebo was associated with more liver cancers among women aged 50–59 years and prior oral contraceptive users.

The higher incidence of liver cancer among women aged 50–59 years or prior oral contraceptive users that were randomized to CEE + MPA should be cautiously interpreted. Chance remains an important consideration, as liver cancer was not a protocol-specified outcome, the overall effect was not statistically significant, and these findings were based on limited events. Age and oral contraceptive use were not randomized, therefore should be viewed as identifying possible treatment-effect heterogeneity rather than causal mechanisms [15]. Recent large prospective and meta-analytic data do not support a strong association between ever oral contraceptive use and liver cancer risk [16]. Thus, these subgroup findings may reflect chance, susceptibility, or other subgroup-associated characteristics rather than causal effects. The concentration of cases among oral contraceptive users does not establish additive “hormonal burden.” Nonetheless, these randomized trial findings may be useful for future cohort studies and meta-analyses.

Liver cancer HRs were directionally different between HT trials, although the CEE-alone confidence interval was wide and compatible with both lower and higher risk. WHI trial findings have differed by formulation, as addition of MPA offset the unfavorable effects of CEE-alone on endometrial and ovarian cancer [17, 18], as well as offset favorable effects of CEE-alone on breast cancer incidence and breast cancer mortality [19], perhaps mediated by apoptotic effects [20]. Thus, formulation-specific liver cancer findings are biologically plausible, as estrogen and progestin signaling can have tissue- and pathway-specific effects, but should be interpreted cautiously given the limited number of events in this analyses.

In separate analyses of each randomized trial, and in a meta-analytic analysis combining the trial-specific HRs, we found that HT was not associated with liver cancer incidence or liver cancer-specific mortality. This neutral analysis finding differs from several observational reports suggesting lower liver cancer risk with HT. In a systematic review and meta-analysis [5], Zhong et al. reported lower primary liver cancer risk among HT users, with directionally similar associations for estrogen-alone and estrogen plus progestin. Similarly, a UK Clinical Practice Research Datalink nested case-control study reported lower liver cancer risk among menopausal hormone therapy users, particularly estrogen-only users [21] and a recent Swedish population-based cohort reported lower hepato-cellular carcinoma risk for both estrogen-only and estrogen-plus-progestogen therapy [22]. These observational findings, generally suggesting approximately 30%–60% lower liver cancer risk among HT users, stand in contrast to the neutral overall WHI HR-estimate and to the CEE + MPA trial finding, which was directionally inconsistent with a large protective effect.

WHI randomized trial findings for HT and female cancers differ from cohort study findings in several areas. In cohort studies, both estrogen-alone and estrogen plus progestin therapies have generally been associated with higher ovarian cancer incidence, whereas only estrogen-alone was significantly associated in the WHI randomized trial [17]. Similarly, cohort studies have shown estrogen-alone to be associated with higher breast cancer incidence and mortality, while WHI randomized trial findings showed significantly lower breast cancer incidence, the protocol-specified primary outcome for safety, and breast cancer mortality with estrogen-alone [11]. This discordance was also seen for coronary heart disease, a protocol-specified primary efficacy outcome in the WHI HT trials; observational studies suggested cardiovascular benefit, whereas WHI trials found no coronary heart disease prevention benefit and early harm with CEE + MPA [23]. These differences may reflect important distinctions in study design, as cohort studies can be influenced by self-selection of healthier women initiating HT, and by depletion of susceptible women who develop early outcomes or discontinue because of intolerable side effects [17, 23]. In contrast, WHI participants were willing to be randomly assigned, in a double-blind manner, to HT or placebo, and outcomes were centrally adjudicated and analyzed regardless of treatment tolerance or adherence. U.S. population trends support the WHI randomized clinical trials evidence whether they agree or disagree with the observational study results. The sharp decline in hormone therapy use after 2002 was followed by lower incidence of breast and ovarian cancers, higher endometrial cancer incidence, and lower incidence of myocardial infarction, consistent with WHI randomized evidence [24–27].

Mechanisms linking estrogen exposure and liver cancer remain incompletely understood. Experimental models suggest protective effects of estrogen through anti-inflammatory pathways, including IL-6 suppression via activation of estrogen receptors on Kupffer cells [28], but observational data are inconsistent [5, 28]. A 2017 meta-analysis of observational studies (1,795 cases) reported protective associations for menopausal hormone therapy overall (RR, 0.60; 95% CI, 0.37–0.96), though these findings were not significant when stratified by estrogen-only or combined therapy [5]. These inconsistencies highlight the value of randomized trial data, as presented here.

Known viral and non-viral risk factors for liver cancer (chronic hepatitis, cirrhosis, smoking, alcohol, obesity, diabetes, thrombocytopenia) [29] were balanced across WHI randomization groups limiting residual confounding. Reproductive factors (prior menopausal hormone therapy and oral contraceptive use) were also balanced, except for modest differences in oophorectomy in the CEE-alone trial.

In prior WHI reports, CEE plus MPA increased risk of coronary disease, stroke, venous thromboembolism, and invasive breast cancer, with few sustained benefits for chronic disease prevention [30, 31]. While CEE plus MPA reduced colorectal cancer incidence early [32], the finding was not supported with longer follow-up [31]. CEE-alone has shown more favorable profiles particularly in younger women [33] including reduction in breast cancer incidence and breast cancer mortality [11]. The present results extend WHI randomized clinical trial findings regarding menopausal hormone therapy influence on liver cancer, a less common endpoint, and indicate no overall benefit of menopausal hormone therapy. Full presentation of the risks and benefits of menopausal hormone therapy have been recently presented elsewhere [33].

### Strengths and limitations

This study has notable strengths including the randomized trial design among a large and well-characterized cohort, with long-term follow up extending nearly 25 years, centralized adjudication of cancer incidence and outcomes. The availability of two hormone therapy trials allowed separate evaluation of CEE-alone and CEE plus MPA. This study has limitations. Primary liver cancer was not a specified secondary study outcome, and the modest number of incident cases limited statistical power. Study findings only apply to the CEE-alone or CEE plus MPA regimens evaluated; however, long-term randomized clinical trial findings for liver cancer are not available for other hormone therapies.

## CONCLUSIONS

In randomized clinical trial settings, menopausal hormone therapy did not alter overall liver cancer incidence and liver cancer specific mortality. However, higher risks were observed with CEE plus MPA among younger women regardless of oral contraceptive use. These findings argue against menopausal hormone therapy for liver cancer prevention and highlight subgroups warranting further study.

## Supplementary Material

Supplemental Material

**Supplementary information** The online version contains supplementary material available at https://doi.org/10.1038/s41416-026-03621-9.

## Figures and Tables

**Table 1.** Baseline Characteristics of Participants in the Women’s Health Initiative Trials of Menopausal Hormone Therapy

Characteristics	CEE-alone Trial				CEE + MPA Trial			
	CEE-alone (n = 5310)		Placebo (n = 5429)		CEE + MPA (n = 8506)		Placebo (n = 8102)	
	N	%	N	%	N	%	N	%
Age at screening, y, mean (SD)	63.6	(7.3)	63.6	(7.3)	63.2	(7.1)	63.3	(7.1)
Age group at screening, y, %								
50–59	1639	30.9	1674	30.8	2837	33.4	2683	33.1
60–69	2386	44.9	2465	45.4	3854	45.3	3655	45.1
70–79	1285	24.2	1290	23.8	1815	21.3	1764	21.8
Hispanic^a^, %	353	6.7	361	6.7	519	6.1	461	5.7
Race^a^, %								
American Indian/Alaska Native	24	0.5	25	0.5	24	0.3	23	0.3
Asian/Pacific Islander	86	1.6	78	1.4	204	2.4	172	2.1
Black/African American	775	14.6	823	15.2	534	6.3	569	7.0
White	4234	79.7	4289	79.0	7480	87.9	7106	87.7
Multiracial, other, or not reported	191	3.6	214	3.9	264	3.1	232	2.9
College degree or higher, %	1217	23.2	1327	24.6	2915	34.4	2839	35.3
Body mass index, kg/m^2^, median [IQR]	29.2	[8.1]	29.2	[7.8]	27.5	[7.5]	27.5	[7.4]
Body mass index group, kg/m^2^, %								
Normal, < 25	1110	21.0	1096	20.3	2579	30.4	2479	30.8
Overweight, 25- < 30	1798	34.0	1915	35.5	2992	35.3	2835	35.2
Obese, ≥ 30	2375	45.0	2385	44.2	2899	34.2	2737	34.0
Waist circumference, cm, mean (SD)	91.7	(14.0)	91.5	(13.7)	88.0	(13.7)	88.0	(13.8)
Smoking, %								
Never	2723	51.9	2705	50.4	4178	49.6	3999	50.0
Past	1986	37.8	2090	38.9	3362	39.9	3157	39.5
Current	542	10.3	571	10.6	880	10.5	838	10.5
Alcohol consumption, %								
Never	718	13.7	737	13.7	972	11.5	938	11.7
Past	1277	24.3	1270	23.6	1427	16.9	1380	17.2
Current	3251	62.0	3379	62.7	6042	71.6	5711	71.1
Age at menarche, y, mean (SD)	12.6	(1.5)	12.6	(1.6)	12.7	(1.5)	12.7	(1.5)
Oral contraceptive use, %	2053	38.7	2052	37.8	3695	43.4	3447	42.5
Parity, %								
Never given birth	491	9.3	463	8.6	860	10.2	833	10.3
Given birth once	381	7.2	445	8.2	690	8.1	661	8.2
Given birth >= 2 times	4398	83.5	4487	83.2	6919	81.7	6572	81.5
Age at menopause, y, mean (SD)^b^	44.5	(7.6)	44.4	(7.7)	50.0	(4.8)	50.0	(4.7)
Bilateral oophorectomy, %	1938	39.5	2111	42.0	29	0.3	24	0.3
Prior MHT use, %	2541	47.9	2659	49.0	2229	26.2	2080	25.7
History diabetes or hyperglycemia, %	502	9.5	520	9.6	488	5.7	470	5.8
History of liver disease^c^, %	133	2.5	127	2.3	202	2.4	177	2.2
History of liver cancer, %	0	0.0	0	0.0	0	0.0	0	0.0
Platelet count, Kcell/uL, median [IQR]	246.0	[73.0]	246.0	[75.0]	243.0	[72.0]	242.0	[70.0]

a Ethnic group and race were self-reported by participants. Multiracial participants self-identified with more than one race. Participants self-identified as ‘other.’

b Some components not assessed when trial began, so data is missing on 15.0% and 8.6% of participants in the CEE-alone and CEE + MPA trials, respectively.

c Includes chronic active hepatitis, cirrhosis, or yellow jaundice.

## Data Availability

Information about data sharing for the Women’s Health Initiative can be found at https://www.whi.org/doc/WHI-Data-Sharing-Statement.pdf.
