# Supplementary material for: Menopausal hormone therapy and primary liver cancer: long-term follow-up of the women’s health initiative randomized trials
Source: Br J Cancer. Author manuscript; Available in PMC 2026 Sep 23. (PMC13599497; doi:10.1038/s41416-026-03621-9)
Supplement: Supplementary file 1 [file NIHMS2210973-supplement-Supplemental_Material.docx]

**Supplemental Material**

**Supplemental Table 1. Distribution of tumor characteristics for liver cancer by randomization group**

|  | **CEE-alone Trial (n=26^a^)** | | | |  | **CEE + MPA Trial (n=48 ^a^)** | | | |
| --- | --- | --- | --- | --- | --- | --- | --- | --- | --- |
| **Tumor characteristics** | **CEE-alone**  **(n=11)** | | **Placebo**  **(n=15)** | |  | **CEE+MPA**  **(n=30)** | | **Placebo**  **(n=18)** | |
|  | **N** | **%** | **N** | **%** |  | **N** | **%** | **N** | **%** |
| **Tumor characteristics available ^a^** | 4 | 36.4 | 13 | 86.7 |  | 20 | 66.7 | 12 | 66.7 |
| **Histology ^b^** |  |  |  |  |  |  |  |  |  |
| Hepatocellular carcinoma | 1 | 25.0 | 7 | 53.8 |  | 11 | 55.0 | 8 | 66.7 |
| Intrahepatic cholangiocarcinoma | 1 | 25.0 | 6 | 46.2 |  | 4 | 20.0 | 4 | 33.3 |
| Other, specified | 2 | 50.0 | 0 | 0 |  | 5 | 25.0 | 0 | 0.0 |
| **Stage ^b^** |  |  |  |  |  |  |  |  |  |
| Local | 1 | 25.0 | 6 | 46.2 |  | 7 | 35.0 | 3 | 25.0 |
| Regional | 0 | 0.0 | 1 | 7.7 |  | 3 | 15.0 | 2 | 16.7 |
| Distant | 3 | 75.0 | 3 | 23.1 |  | 5 | 25.0 | 3 | 25.0 |
| Unknown/not done/missing | 0 | 0.0 | 3 | 23.1 |  | 5 | 25.0 | 4 | 33.3 |
| **Grade ^b^** |  |  |  |  |  |  |  |  |  |
| Well differentiated | 0 | 0.0 | 2 | 15.4 |  | 2 | 10.0 | 1 | 8.3 |
| Moderately differentiated | 2 | 50.0 | 2 | 15.4 |  | 2 | 10.0 | 3 | 25.0 |
| Poorly differentiated | 0 | 0.0 | 2 | 15.4 |  | 4 | 20.0 | 0 | 0.0 |
| Unknown/not done/missing | 2 | 50.0 | 7 | 53.8 |  | 12 | 60.0 | 8 | 66.7 |

**^a^** Tumor characteristics were not available for n = 9 (34.6%) participants in the CEE-alone trial (7 CEE vs 2 placebo; 63.6 vs 13.3%) as ascertainment and adjudication of liver cancer was exclusively based on report of death. Likewise, tumor characteristics were not available and exclusively based on report of death for n = 16 participants in the CEE+MPA trial (10 CEE+MPA vs 6 placebo; 33.3 vs 33.3%). Availability statistically differed between randomization groups for the CEE-alone trial (P = 0.014), but not for the CEE+MPA trial (P > 0.99); P-values based on Fisher’s exact test.

**^b^** Excludes participants whose ascertainment and adjudication of liver cancer was exclusively based on report of death as data are 100% missing.

**Supplemental Figure 1. Association of CEE+MPA with liver cancer incidence by baseline subgroups during cumulative follow-up.**

Hazard ratios (HRs) and 95% confidence intervals (CIs) were estimated using Cox proportional hazard models, which included an interaction term between treatment x subgroup, with baseline hazard functions stratified by age group, dietary modification trial randomization, study period (time-dependent), and subgroup.

^a^ Annualized rates were calculated by dividing the total number of events by total follow-up time in years and expressed as a percentage.

^b^ Difference in estimated absolute excess risks (HT minus placebo) for 10,000 person-years.

^C^ P-value corresponds to a two-sided stratified score (log-rank) test of the main effect or interaction. Interactions with age and BMI groups were assessed with a 1–degree-of-freedom test for linear trend, with each subgroup assigned an integer value of 0, 1, or 2.
